# Volatile Anesthesia Activates Renal Sympathetic Nerves to Reduce Renal Excretory Function: Implications for Surgically-Induced Acute Kidney Injury

**DOI:** 10.1093/function/zqab056

**Published:** 2021-11-09

**Authors:** John W Osborn, Arthur Cruz-Lynch

**Affiliations:** Department of Surgery; The Graduate Program in Integrative Biology and Physiology, University of Minnesota, Minneapolis, MN 55455, USA; The Graduate Program in Integrative Biology and Physiology, University of Minnesota, Minneapolis, MN 55455, USA

## A Perspective on “Role of Renal Sympathetic Nerve Activity in Volatile Anesthesia's Effect on Renal Excretory Function”

### The Problem: Oliguria During Surgery and Anesthesia

A marked reduction in urine output, oliguria, is a common occurrence in patients undergoing surgical procedures under volatile anesthesia.^1^ Prolonged periods of oliguria or acute increases in serum creatinine are important criteria for identifying and staging acute kidney injury (AKI) following anesthesia and surgery.^2^ The cause of oliguria during anesthesia and surgery is unknown, but anesthesia-induced reductions in renal perfusion pressure and mechanical ventilation of the patient have been proposed.^3^ An increase in plasma arginine vasopressin (AVP), which increases tubular water reabsorption, thereby decreasing urine production, has been suggested as the mechanism of reduced renal excretory function under volatile anesthesia,^3^ but this hypothesis has not been tested. A clear understanding of the mechanism of oliguria during surgery and anesthesia is important since preventing it could reduce the incidence of AKI and associated complications (ie, pulmonary edema, cardiorenal syndrome, uremic encephalopathy, and hepatic inflammation).^4^

Another explanation is that volatile anesthetics have direct or indirect effects on renal function that markedly reduce urine output. For example, although volatile anesthetics are generally thought to suppress the overall output of the sympathetic nervous system, isoflurane has been reported to increase sympathetic nerve activity (SNA) to the kidneys specifically.^5^ The kidneys are richly innervated by sympathetic nerves which, when activated, reduce urinary sodium excretion via three mechanisms; (1) constriction of the afferent renal arteriole, thereby reducing glomerular filtration rate, (2) stimulation of renin release from juxtaglomerular cells resulting in increased plasma angiotensin II and aldosterone to increase sodium reabsorption, and (3) the direct effect of sympathetic nerves to increase tubular sodium reabsorption.^6^ Therefore, an increase in renal SNA (RSNA) can markedly reduce urine output by both reducing the filtered load of sodium into the nephron and increasing tubular sodium reabsorption.

### The Hypothesis

Taavo and colleagues hypothesize that oliguria observed in surgical procedures conducted using volatile anesthetics is due to an increase in RSNA.^7^ If this is correct, then reduced urine output should be associated with changes in renal function known to be influenced by renal sympathetic nerves.

### The Approach

To test this hypothesis, these investigators conducted an elegant series of clinical and preclinical studies to determine whether the commonly used volatile anesthetic, sevoflurane, reduced urine output by increasing RSNA.

In the first study, indices of renal function were measured before and during sevoflurane anesthesia in eight male children undergoing hypospadias surgery. Compared to the conscious state, surgery and sevoflurane anesthesia resulted in several responses that are all consistent with an increase in RSNA including reductions in urinary flow (−65%) and urinary sodium excretion (−49%) and a 10-fold increase in plasma renin concentration. Importantly, there was no change in plasma AVP or free water clearance. Taken together, these results suggest the oliguria was due to increased tubular reabsorption of sodium rather than water.

Since RSNA cannot be directly measured in humans, a second study was conducted in ewes that were chronically instrumented to measure arterial pressure, renal blood flow, and RSNA while simultaneously measuring renal excretory function. Measurements were made first in the conscious state and then under sevoflurane anesthesia. This is an exceptionally impressive approach as the direct measurement of SNA in conscious animals is possible in only a handful of laboratories in the world.^8^ As in the children undergoing hypospadias surgery, sevoflurane reduced urine output (−52%) and urinary sodium excretion (−85%). More importantly, RSNA was dramatically increased, whether quantified by burst frequency (+105%) or burst incidence (+51%) under sevoflurane compared to the conscious state. In addition, sevoflurane resulted in significant renal vasoconstriction. Cardiorenal function and RSNA were also measured 2 h after an intravenous fluid load in the conscious and anesthetized state. This volume loading protocol was conducted for two reasons. First, it would establish whether increased RSNA was simply due to hypovolemia associated with the procedure. Second, it would directly address whether administration of intravenous fluids to treat oliguria was effective. The results clearly demonstrate that the increase in RSNA is not secondary to hypovolemia and treating oliguria by intravenous fluid therapy was ineffective.

A third study directly tested the hypothesis that sevoflurane-induced oliguria was dependent on renal nerves by comparing renal function in sheep with intact renal nerves to those that had undergone surgical renal denervation (RDN). Urine flow rate and sodium excretion were higher in sevoflurane-anesthetized RDN sheep compared to those with intact renal nerves. However, there were no differences in these variables in the conscious state. Similarly, renal blood flow was higher in sevoflurane-anesthetized RDN sheep compared to those with intact renal nerves, but no differences were observed in the conscious state. Taken together, these findings are consistent with the hypothesis that intact renal nerves are required for the sevoflurane-induced alterations in renal hemodynamics and, more importantly, oliguria.

### Summary and Significance

The study by Taavo and colleagues^7^ provides powerful evidence that oliguria observed with volatile anesthetics is the result of increased RSNA rather than increased plasma vasopressin or hypotension. Beyond establishing the mechanisms mediating reduced urine output during anesthesia with volatile anesthetics, the study is clinically significant in that it clearly explains why fluid resuscitation during anesthesia in many patients is ineffective in improving urine output and preventing postoperative AKI. Furthermore, although this was not the intent of this investigation, this study highlights the powerful influence of renal sympathetic nerves on renal function which has important implications for the recent development of catheter-based renal nerve ablation for the treatment of drug-resistant hypertension in humans.^9^

What remains unclear however is the mechanism by which volatile anesthetics increase RSNA and how this neurally mediated oliguria could be treated using pharmacological antagonists to block the renal responses to increased RSNA. However, such an approach would be complicated by the systemic actions of alpha and beta blockers which would reduce arterial pressure. Nonetheless, the study by Taavo and colleagues clearly illustrates that volatile anesthetics should be avoided in surgical patients already at risk for AKI.
